# A Comparison of the Performance of Horses from the International WBFSH Rankings in Dressage, Jumping and Eventing Based on Their Sex, Age, Proportion of Thoroughbred Genes and Affiliation to the Studbook

**DOI:** 10.3390/ani16101509

**Published:** 2026-05-14

**Authors:** Eva Sobotková, Dana Kuřitková, Paulina Zeliszewska-Duk, Vladimír Mikule

**Affiliations:** 1 Department of Animal Breeding, Mendel University in Brno, Zemědělská 1, 613 00 Brno, Czech Republicvladimir.mikule@mendelu.cz (V.M.); 2 Department of Horse Breeding and Utilization, University of Life Sciences in Lublin, Dobrzańskiego 35, 20-262 Lublin, Poland; paulina.zeliszewska@up.lublin.pl

**Keywords:** equine, performance, equestrian sport, mare, stallion, gelding

## Abstract

The aim of the research was to assess how the selected factors could influence the performance of the best horses in dressage, jumping and eventing competitions. The research was based on the Studbook Rankings published by the World Breeding Federation for Sport Horses from 2019 to 2024. Our analysis shows a statistically significant effect of each of the observed factors in at least one discipline. The leading factor in all disciplines was studbook affiliation. The Belgian warmblood, German Sport Horse, Hanoverian, Royal Dutch Sport Horse, Oldenburg and Westphalian studbooks are represented among the significantly best studbooks in all three disciplines. Geldings were statistically highly demonstrably the best in jumping. In eventing, both geldings and stallions were highly significantly better than mares. Significantly, the highest performance was achieved by 11-year-old horses in jumping and by 15-year-old horses in eventing. We have demonstrated a statistical effect of the proportion of thoroughbred genes on performance in dressage. Statistically better performance in a selected elite population of dressage horses was achieved by horses with 20% to 50% thoroughbred genes. Our results confirm the necessity of selecting sport horses based on specialized performance tests and the need for different breeding programs for success in individual equestrian sports.

## Simple Summary

The horse is the only animal in the world that is bred for use in top-level sport up to the Olympic Games level. To maintain progress in breeding sport horses, it may prove useful to analyze the impact that various factors may have on the capabilities of horses. In this article, we show how age, sex, as well as the proportion of thoroughbred genes in a horse’s pedigree and affiliation to a studbook influence the performance of horses in dressage, jumping and eventing competitions at the highest international level. Our results show that the importance of the monitored factors on the performance of the best horses from the international ranking varies with respect to the individual disciplines. Even though the only common statistically significant indicator of the best horses throughout the disciplines is the affiliation to a studbook, we found that the dressage performance of horses with different proportions of thoroughbred genes in their pedigree differs statistically significantly, and the performance of horses in the jumping and eventing rankings is significantly influenced by the sex and age of the horses. Based on this analysis, we believe that we have demonstrated fundamental differences between the best horses in the studied disciplines, from which experts can derive practical breeding measures. But the results of this study of a selected elite population cannot be generalized, and in the future it is necessary to verify these results on unlimited populations.

## 1. Introduction

The horse is the only animal to be bred throughout history not only for its economic and working use but also for its athletic abilities and sporting use up to the level of the Olympic Games [1,2,3].

The three Olympic equestrian sports require different athletic attributes each.

Horses for eventing must be bold, agile, and able to gallop in an energy-efficient manner to meet the speed requirements. Jumping horses must be physically strong and powerful to be able to launch themselves high into the air and clear obstacles over 160 cm. In dressage, the horse must show a larger number of gaits and movements, which requires an advanced level of balance and muscular control, while performing in a progressively uphill posture [4,5].

The fact that a specialized breeding program can be more effective if it defines two or more separate breeding goals for each discipline has been verified by several scientific studies [6,7,8,9]. Therefore, the need to breed populations specialized in different equestrian disciplines has already been incorporated into breeding directions by most sport horse studbooks (Royal Dutch Sport Horse (KWPN), Hanoverian horse, Belgian warmblood, Oldenburg horse, Swedish warmblood, etc.).

In addition to the different requirements for breeding the ideal horse for individual disciplines, the estimated heritability values of performance traits also differ. The heritability for the jumping performance of the KWPN fluctuates from 0.08 to 0.28, and from 0.07 to 0.25 for dressage, depending on other variables factored into their calculation model [10]. Similar results were also obtained for Swedish warmblood horses (dressage *h*^2^ = 0.16, jumping *h*^2^ = 0.28) [11]. For performance in eventing competitions, the heritability estimate values were lower: 0.11–0.17 [12] or 0.16 [13]. At the advanced levels of eventing tests jumping showed the highest heritability, followed by dressage (0.171) and then cross-country (0.039) [14]. These relatively low values of *h*^2^ reflect the large non-genetic effect on differences in performance in the aforementioned disciplines [15].

The most frequently monitored factors in performance analyses of sport horses are, in various combinations: age, sex, breed, sire, rider, training and proportion of thoroughbred genes [10,16,17,18,19,20,21,22,23,24,25,26,27,28,29].

The results of proving or disproving the influence of individual factors vary from study to study, depending on the disciplines, the breeds, the countries studied, and the levels of performance tested.

In this study, the influence of the sire could not be assessed due to the small number of offspring of each sire, and the influence of the rider was not tested due to the small number of horses and the difficulty of estimation, as there is significant imbalance in the analytical models. Furthermore, previous research [25,30,31,32,33] has shown that rider influence is relevant at lower-level competitions and that rider influence decreases with increasing difficulty of the competition and with increasing age of the horses in both dressage and jumping. At the advanced level of international competitions, only the experienced horses over 6 years old and riders with extensive experience, nominated by national federations to represent them, compete.

The aim of our study is to objectively assess the relevancy of the selected factors on the performance of the most successful horses in all three Olympic equestrian disciplines together, and to evaluate the similarities and differences in the best horses within these disciplines.

## 2. Materials and Methods

The aim of our analysis was to objectively evaluate the best horses competing in international dressage, jumping and eventing with regard to their studbook, proportion of thoroughbred (TB) genes in the pedigree as well as age, sex and sport season. The research was based on the Studbook Rankings published by the World Breeding Federation for Sport Horses (WBFSH) [34]. These databases include 6 horses within each studbook that achieved the best results at the highest-level international competitions. This information is publicly accessible through the WBFSH website and performance points are calculated using validated International Federation for Equestrian Sports (FEI) results [35]. Our research included the results from the WBFSH Studbook Rankings in dressage, jumping and eventing from 2019 to 2024. Only studbooks that were represented by at least 20 entries during the monitored years were included in the analysis. A list of all monitored studbooks and their abbreviations is provided at the end of the article.

The databases consisted of 3815 horses (691 horses in dressage, 1488 horses in jumping, and 1636 horses in eventing) in total. The following data were extracted from the WBFSH and FEI databases: horse name, identification number, age in years (from 6 to 22 years old), sex (stallion, mare, or gelding), studbook affiliation (the formal registration of a horse to an official breed registry: 22 in dressage, 45 in jumping, and 50 in eventing; the list of all evaluated studbooks is given in the list of abbreviations), and ranking points [34,35]. During its career, a horse may have appeared in the competition rankings in several years. Annually, its performance points were calculated from zero according to its current starts in competitions, and in terms of age, such a horse belonged to a different group. Therefore, such results are considered relevant because they reflect the real situation in a given year of the ranking. Previous results do not give a horse an advantage and can generally be judged each year independently without reference to the individual horse. However, this is a limitation of the study design. The proportion of TB genes was calculated according to HorseTelex Pedigree [36]. The exact values of the proportion of TB genes over 9 generations were used to evaluate the descriptive statistics of TB genes for each discipline.

The data were processed in Microsoft Excel 2016 and the statistical analyses were performed in Statistica 2012 (version 14.0.0.15). For the basic evaluation of the database, we used the descriptive statistics (mean, mode, median, minimum, maximum and standard deviation) of ranking points and the proportion of TB genes. The generalized linear model (GLM) method (multifactor analysis with fixed effects) was used to assess the influence of the selected factors. Age, sex, studbook, sport season and the proportion of TB genes in the pedigree were treated as the main effects in the statistical analyses for each of the disciplines studied. *Y* had an approximately normal distribution (based on QQ plot assessment), so we did not use a logarithmic link or any other data transformation due to the large number of observations. The homogeneity of variances between groups of fixed factors was tested by Bartlett’s test.

To evaluate the significance of the proportion of TB genes, we chose a categorical factor instead of a continuous variable because we want to compare the performance results of groups with different proportions of TB genes in the disciplines under study. The aim of the analysis was not to evaluate the regression relationship between the proportion of TB genes and performance, as this would not allow a detailed comparison of the performance trend across disciplines. In order to use the proportion of TB genes as an effect and assess differences between disciplines, groups by 10% proportion were created (up to 10 group: 0–10.00% TB genes; up to 20 group: 10.01–20.00% TB genes; up to 30 group: 20.01–30.00% TB genes; up to 40 group: 30.01–40.00% TB genes; up to 50 group: 40.01–50.00% TB genes; up to 60 group: 50.01–60.00% TB genes; up to 70 group: 60.01–70.00% TB genes; up to 80 group: 70.01–80.00% TB genes; up to 90 group: 80.01–90.00% TB genes; up to 100 group: 90.01–100.00% TB genes).

Smaller groups and outliers were retained and we did not merge any data to avoid distorting the real situation in the evaluation. In the results, we point out specific situations where the reliability of the results is reduced due to small representation. In order to maintain objectivity, graphs with standard errors of differences are also attached, which most accurately depict individual situations.

The following model equation was used:

y_ijklmn_ = µ + b_i_ + g_j_ + a_k_ + t_l_ + s_m_ + e_ijklmn_,

where:

y_ijklmn_ is the evaluated quantity;

µ is the overall average of the set;

b_i_ in the first analysis is the fixed effect of the i-th studbook;

g_j_ is the fixed effect of the j-th sex;

a_k_ is the fixed effect of the k-th age;

t_l_ is the fixed effect of the l-th group based on the proportion of TB genes;

s_m_ is the fixed effect of the m-th group based on sport season;

e_ijklmn_ is the residual error.

Subsequently, the method of multiple comparisons by Scheffé’s test was used to define significant differences caused by the individual effects. Scheffé’s test is a post hoc test used in the GLM method. After the GLM had been run and a significant F-statistic had been obtained, Scheffé’s test was then performed to find out which pairs of means were significant. The methods used were selected based on the availability of data and the results of studies by other authors monitoring the performance of sport horses. This statistical model was used in connection with our previous research, where we tested the population of horses in international jumping competitions based on the same factors. By maintaining a similar procedure, the new results can be compared with the previous analysis of jumping competitions, where we tested the entire population.

No Animal Care and Use Committee approval was obtained for this study because the data were obtained from an existing database publicly accessible on the WBFSH website [34].

## 3. Results

### 3.1. Evaluation of the Data Set

Table 1 shows the achieved range of performance points for the individual disciplines. The dressage data set had the fewest horses evaluated in the WBFSH ranking and the set also had the highest variability; furthermore, unlike jumping and eventing, achieving a minimum of 1 point was not enough for a horse to be included. Due to the different methodologies for calculating performance points, a direct comparison could not be calculated, as an appropriate conversion to a uniform performance evaluation system would be necessary.

#### 3.1.1. Evaluation of the Data Set Based on Sex

The proportions of the representation of each sex in the best horse scores in all the disciplines studied clearly showed the predominance of geldings (Table 2). Mares are the least represented in dressage and there were the fewest stallions among the best horses in eventing.

#### 3.1.2. Evaluation of the Data Set Based on Age

**Figure 1:**
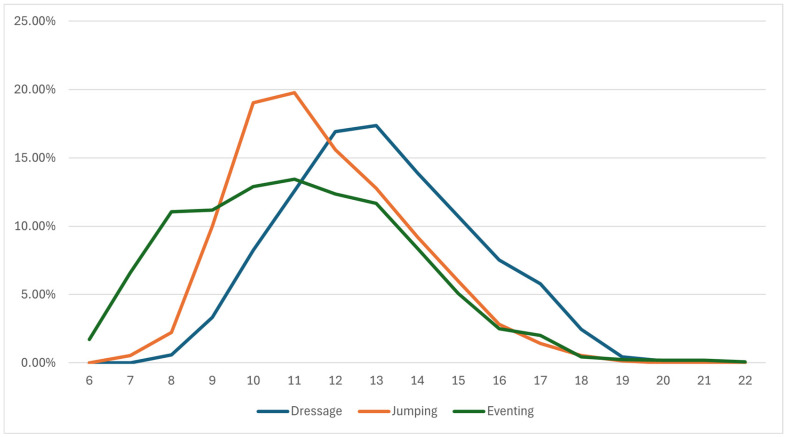
A representation of horses of different ages among the most successful horses in equestrian disciplines.

The frequency of horses of different ages varied within disciplines, as shown in Figure 1. Jumping and dressage had a very similar age composition curve of the best horses, only shifted by two years of the horses’ age. The most balanced representation of horses was in eventing. More than 10% of horses were between 8 and 13 years old, with the number of older horses decreasing rapidly. Only a few horses under the age of 8 scored among the best in jumping. Meanwhile, 10- and 11-year-old horses prevailed, and then the number of horses represented decreased with age. In comparison, the onset of the number of horses in dressage was slower up to the age of 12, with the 13-year-old horses being the most frequently represented age. The relative numbers decreased with age again.

#### 3.1.3. Evaluation of the Data Set Based on the Proportion of Thoroughbred Genes in Pedigree

A comparison of the population of the best horses in the rankings of the monitored disciplines in terms of the proportion of TB genes in pedigree shows a clear difference between dressage and other disciplines (Table 3). On the other hand, there are horses with zero TB genes and the mean of their entire population is also the lowest (28.47%) by a significant margin.

**Figure 2:**
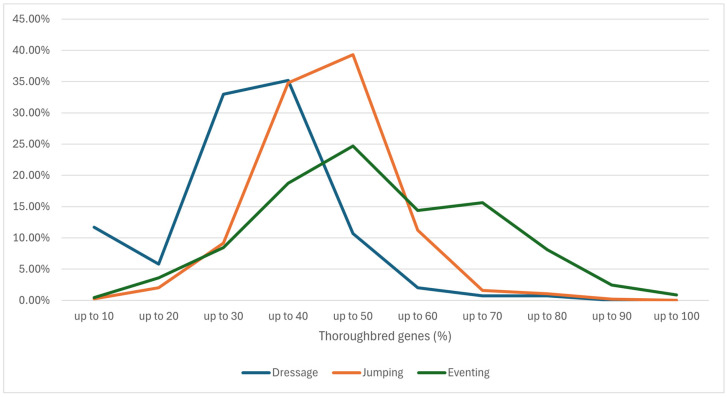
A representation of the groups of horses with different proportions of TB genes among the most successful horses in the WBFSH rankings.

The different representation of TB genes in horses within the monitored populations can be seen in detail in Figure 2. Dressage and jumping have a similar curve with a high proportion of only two groups of horses; the curves are shifted by 10% difference and meet at the proportion of 70% TB genes. The 10% proportion of dressage horses with a minimum proportion of thoroughbred genes in origin is mainly caused by the Purebred Spanish horse (PRE) and Lusitano (LUSIT) breeds.

The eventing curve is more even with two peaks at 50% and 70% of TB genes. If we ignore the extreme values, then, unlike the previous disciplines, the proportion of individual groups is more even over a larger range of values of the proportion of TB genes.

#### 3.1.4. Evaluation of the Data Set Based on the Studbook Affiliation

During the six years that our research focused on, 52 different studbooks appeared in the dressage rankings, 64 in jumping, and, with the largest number of horse breeds evaluated, eventing (67) [34]. Since our research was focused only on those studbooks with at least 20 entries over the monitored period, the numbers decreased to 22 in dressage, 45 in jumping, and 50 in eventing. However, not all of these studbooks were represented by the maximum possible number of horses in all years. The numbers of studbooks that reached the maximum possible number of horses were 11 in dressage, 30 in jumping, and 26 in eventing.

Overall, only eight studbooks combined all disciplines and the maximum number of horses representing them in the rankings (Belgian warmblood—BWP; German Sport Horse—DSP; Hanoverian—HANN; Royal Dutch Sport Horse—KWPN; Oldenburg—OLDBG; Rhenish Warmblood—RHEIN; Swedish warmblood—SWB; and Westphalian—WESTF). From these results, and in connection with the total registered numbers of horses in the disciplines, conclusions can be drawn about their popularity and the related current focus of horse breeding.

### 3.2. Statistical Analysis (GLM and Scheffé’s Test)

The generalized linear model (GLM) method (multifactor analysis with fixed effects) was used to assess the relevancy of the selected factors. Subsequently, the method of multiple comparisons by Scheffé’s test was used to define significant differences caused by the individual effects. Our GLM analysis shows a statistically significant effect of each of the observed factors in at least one discipline (Table 4). The leading factor in all disciplines was studbook affiliation. The significant influence across all disciplines was the sports season. The influence of the proportion of TB genes in the horses’ ancestry on performance was statistically highly conclusively evaluated only in dressage. On the other hand, the influence of the age and sex of the horses was confirmed in jumping and eventing. The results of this study of a selected population cannot be generalized. Further research based on the analysis of entire populations of horses competing in the disciplines studied will be necessary to objectively assess the relevancy of the observed factors.

#### 3.2.1. Effect of Sport Season (By Scheffé’s Test)

A statistically significant difference between the results of individual sports seasons was demonstrated only for 2020 compared to all other seasons. During the entire monitored period, there was no change in the calculation methodology, and therefore we believe that the only reason for this result is the COVID-19 pandemic. It caused the cancelation of some competitions during the 2020 season, which had an impact on the calculation of horses’ performance points.

#### 3.2.2. Effect of Sex (By Scheffé’s Test)

Geldings were statistically highly significantly the best in jumping compared to stallions and mares. In eventing, both geldings and stallions were highly significantly better than mares, which achieved the lowest number of performance points, but no statistical difference was demonstrated between the performance of geldings and stallions. Statistical analysis did not show any effect of sex on performance based on the dressage horse ranking, and therefore we can only conclude, based on average values (Table 5), that stallions achieved the best average dressage results while mares again achieved the lowest average performance.

#### 3.2.3. Effect of Age (By Scheffé’s Test)

In the dressage ranking, no statistical effect of age on the performance of horses was demonstrated. Based on the average values, horses aged ten years and older scored the most performance points (1651.37). Horses aged 12 to 18 achieved balanced performance with minor deviations in the order of tens of points. Horses aged 19 and older scored the fewest points (1277.33 and under).

Statistically highly significant differences between horses of different ages in jumping and eventing are shown in Table 6. The reliability of the results of the oldest age groups in individual disciplines is impaired by their minimal representation.

**Figure 3:**
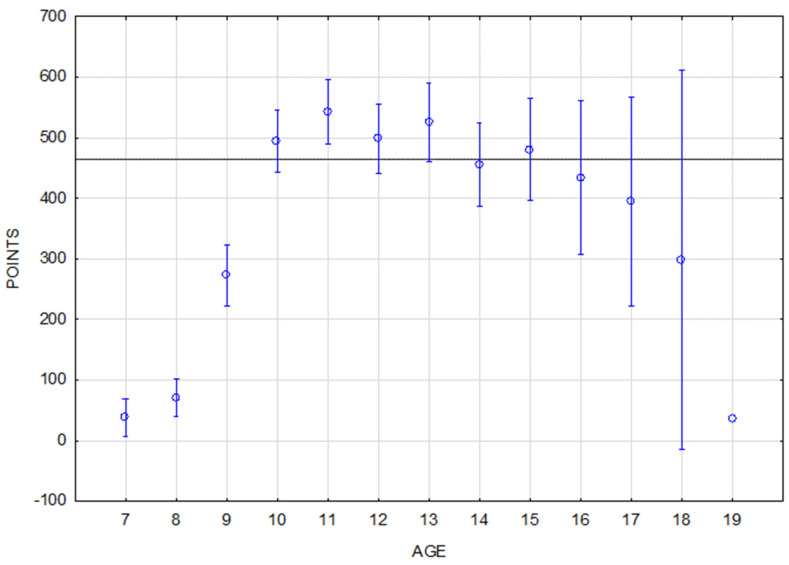
A performance summary of the jumping horses according to age. The horizontal line denotes the mean rating of the monitored population. The vertical bars represent the standard errors of differences (confidence interval 0.95).

A clear steep increase in performance points from the youngest 7-year-old horses to the 10-year-old horses can be seen in the jumping data set (Figure 3). The highest performance was achieved by 11-year-old horses. Scores decreased slightly between ages 11 and 15 with 15-year-old horses being the last group with above-average results. Performance dropped significantly below the average of the observed population only in 16-year-old and older horses.

**Figure 4:**
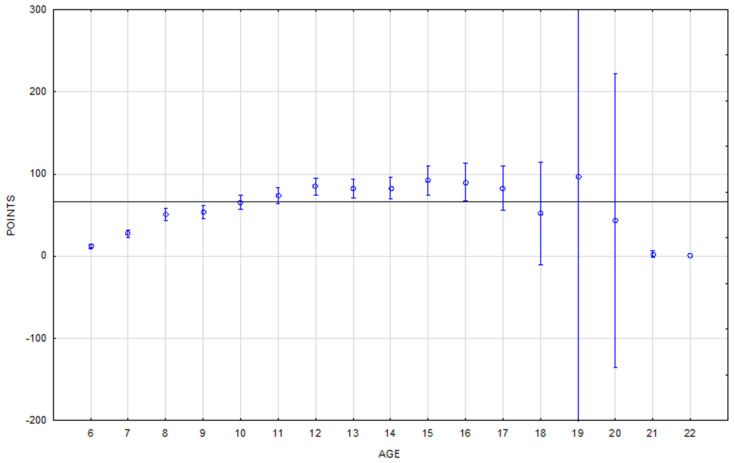
A performance summary of the eventing horses according to age. The horizontal line denotes the mean rating of the monitored population. The vertical bars represent the standard errors of differences (confidence interval 0.95).

The increase in horse performance with age in the eventing rankings is much less dramatic (Figure 4). Horses achieved above-average performance from eleven to seventeen years of age, with the maximum performance points achieved by 15-year-old horses.

#### 3.2.4. Effect of the Proportion of Thoroughbred Genes in Pedigree (By Scheffé’s Test)

The only discipline where we have demonstrated a statistical effect of the proportion of TB genes on performance is dressage (Table 7). Statistically highly significantly better performance was achieved by horses with between 20 and 50% TB genes, as well as by the group with a minimal proportion of thoroughbred genes in the pedigree (up to 10%). The four statistically most successful groups are also the only groups with above-average performance.

**Figure 5:**
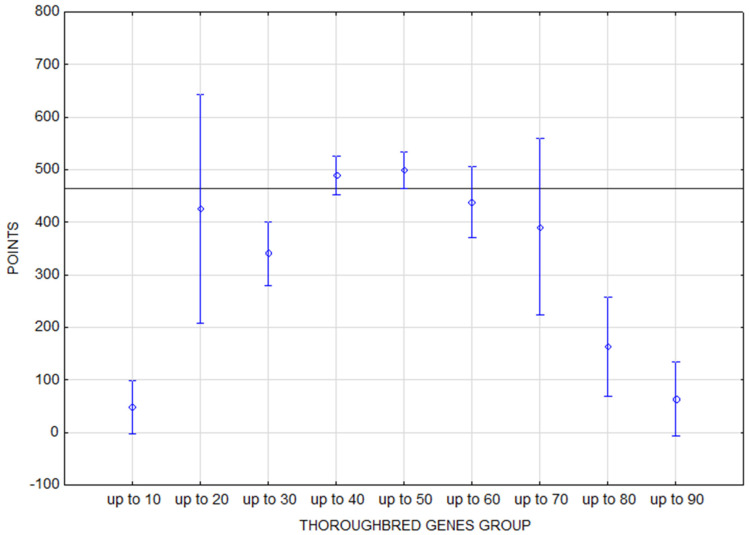
A performance summary of the jumping horses according to the proportion of TB genes. The horizontal line denotes the mean rating of the monitored population. The vertical bars represent the standard errors of differences (confidence interval 0.95).

In the analysis of the jumping rankings, only horses with up to 30% and up to 50% TB genes in the pedigree achieved above-average performance, but the statistical relevancy of the proportion of TB genes was not demonstrated. The performance trend corresponds to a parabolic curve with the lowest performance of the marginal groups with the minimum and maximum representation of thoroughbreds in their pedigree (Figure 5). The performance deviation from the overall trend in horses up to 20% TB genes is caused by the exceptional results of only one horse (King Edward). This group in the observed jumping population represents only 2% of horses and the only gelding mentioned achieved an average performance of 1723.75 points over the observed years. If we eliminate this unique horse from the calculation, the performance of all other horses in this group has an average rating of 226.65 points, which corresponds exactly to the population trend. Thanks to this example, it is clear that the results of groups of horses with minimal representation in the database can be influenced by the outstanding performances of the outliers, and it is necessary to mention such cases and evaluate them individually.

**Figure 6:**
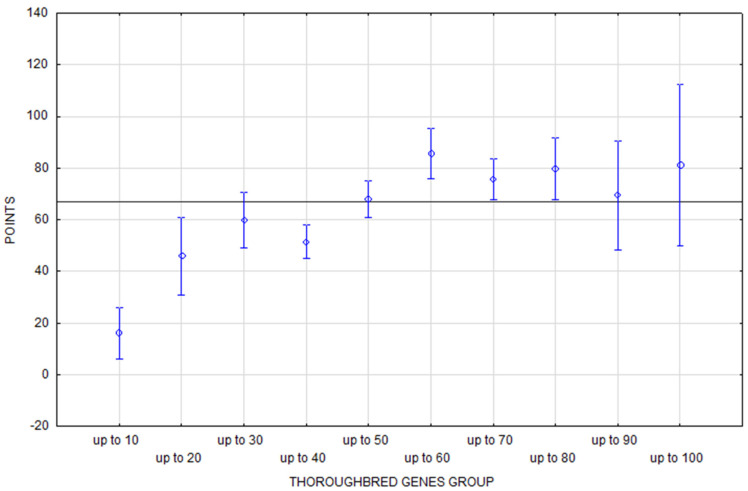
A performance summary of the eventing horses according to the proportion of TB genes. The horizontal line denotes the mean rating of the monitored population. The vertical bars represent the standard errors of differences (confidence interval 0.95).

The performance trend based on the eventing ranking results is diametrically different (Figure 6). The results are also statistically inconclusive, but based on the average values it is clear that only the groups of horses with more than 50% of TB genes (50% to 100%) achieved above-average performance. However, here too we must be aware that the results of up to 90% and up to 100% of the group may be distorted due to the small relative minimal representation (a total of 3.30% of the horses).

#### 3.2.5. Effect of Studbook Affiliation (By Scheffé’s Test)

Studbook affiliation had a statistically highly significant effect on the performance of horses in the WBFSH rankings of all monitored disciplines (Table 4). The most successful studbooks in the rankings of individual disciplines are listed in the summary (Table 8). Only six breeds (BWP, DSP, HANN, KWPN, OLDBG, and WESTF) are represented among the best studbooks in all three disciplines.

Statistically Proven Results of Dressage Performance Analysis (*p* ˂ 0.01)

A list of all studbooks and their abbreviations is provided at the end of the article.

- KWPN showed significantly better performance than PRE, PSH, NZHS, FWB, AWO, BHHS, BWP, TRAK, RHEIN, SWB, ASH, KWPN NA, CH, PZHK, and HOLST.
- OLD, HANN showed significantly better performance than PRE, PSH, NZHS, FWB, AWO, BHHS, TRAK, RHEIN, SWB, ASH, KWPN NA, CH, PZHK, and HOLST.
- WESTF showed significantly better performance than PRE, PSH, NZHS, FWB, AWO, BHHS WP, TRAK, RHEIN, SWB, ASH, KWPN NA, CH, PZHK, and HOLST.
- DWB showed significantly better performance than PRE, PSH, NZHS, FWB, AWO, BHHS, RHEIN, SWB, ASH, KWPN NA, CH, PZHK, and HOLST.
- DSP showed significantly better performance than PRE, PSH, NZHS, FWB, AWO, BHHS, BWP, ASH, KWPN NA, CH, PZHK, and HOLST.
- LUSIT showed significantly better performance than NZHS, FWB, AHS, KWPN NA, and PZHK.
- BWP showed significantly better performance than NZHS, FWB, ASH, KWPN NA, and PZHK.

A detailed graph of the performance summary of the dressage horses according to studbook is attached in Supplementary Files.

Statistically Proven Results of Jumping Performance Analysis (*p* ˂ 0.01)

A list of all studbooks and their abbreviations is provided at the end of the article.

- BWP showed significantly better performance than all studbooks except DSP, HANN, HOLST, KWPN, OS, SF, WESTF, and ZANG.
- SF showed significantly better performance than all studbooks except BWP, DSP, HANN, HOLST, ISH, KWPN, OS, WESTF, and ZANG.
- KWPN showed significantly better performance than all studbooks except BWP, DSP, HANN, HOLST, ISH, OS, SF, WESTF, and ZANG.
- ZANG showed significantly better performance than all studbooks except AES, BWP, DSP, HANN, HOLST, ISH, KWPN, OLDBG, OS, SBS, SF, SWB, and WESTF.
- HOLST showed significantly better performance than all studbooks except AES, BWP, DSP, HANN, ISH, KWPN, OLDBG, OS, SBS, SF, SWB, WESTF, and ZANG.
- OS showed significantly better performance than all studbooks except AES, BWP, DSP, HANN, HOLST, ISH, KWPN, OLDBG, RHEIN, SBS, SF, SWB, WESTF, and ZANG.
- WESTF showed significantly better performance than all studbooks except AES, BWP, DSP, HANN, HOLST, ISH, KWPN, OLDBG, OS, RHEIN, SBS, SF, SWB, and ZANG.
- HANN showed significantly better performance than all studbooks except AES, BWP, DSP, HOLST, ISH, KWPN, MECKL, OLDBG, OS, RHEIN, SBS, SF, SWB, WESTF, and ZANG.
- DSP showed significantly better performance than all studbooks except AES, BWP, HANN, HOLST, ISH, KWPN, MECKL, OLDBG, OS, RHEIN, SBS, SF, SWB, WESTF, and ZANG.
- ISH showed significantly better performance than all studbooks except AES, BWP, DSP, HANN, HOLST, KWPN, MECKL, OLDBG, OS, PZHK, RHEIN, SBS, SF, SWB, WESTF, and ZANG.
- SBS showed significantly better performance than all studbooks except AES, BWP, DSP, HANN, HOLST, ISH, MASAF, MECKL, OLDBG, OS, PZHK, RHEIN, SLS, SWB, WESTF, and ZANG.
- SWB showed significantly better performance than all studbooks except AES, BWP, DSP, HANN, HOLST, ISH, MASAF, MECKL, OLDBG, OS, PZHK, RHEIN, SBS, SLS, WESTF, and ZANG.
- AES showed significantly better performance than all studbooks except BE/SIES, BWP, DSP, DWB, ESH, CH, HANN, HOLST, ISH, MASAF, MECKL, OLDBG, OS, PZHK, RHEIN, SBS, SLS, SWB, WESTF, and ZANG.
- OLDBG showed significantly better performance than all studbooks except AES, BE/SIES, BWP, DSP, DWB, ESH, CH, HANN, HOLST, ISH, MASAF, MECKL, OS, PZHK, RHEIN, SBS, SLS, SWB, WESTF, and ZANG.
- RHEIN showed significantly better performance than AA, HSH, ChS, NWB, PSH, SAWHS, and SHBGB.

A detailed graph of the performance summary of the jumping horses according to studbook is attached in Supplementary Files.

Statistically Proven Results of Eventing Performance Analysis (*p* ˂ 0.01)

A list of all studbooks and their abbreviations is provided at the end of the article.

- ISH showed significantly better performance than all studbooks except HANN, HOLST, KWPN, SF, and SHBGB.
- HOLST showed significantly better performance than all studbooks except AES, DSP, HANN, ISH, KWPN, SF, and SHBGB.
- SF showed significantly better performance than all studbooks except AES, DSP, HANN, HOLST, ISH, KWPN, SF, and SHBGB.
- HANN showed significantly better performance than all studbooks except AA, AES, BWP, DSP, HOLST, ISH, KWPN, OLDBG, SF, SHBGB, SWB, and WESTF.
- KWPN showed significantly better performance than all studbooks except AA, AES, BWP, DSP, HANN, HOLST, ISH, OLDBG, SF, SHBGB, SWB, WESTF, and ZANG.
- SHBGB showed significantly better performance than all studbooks except AA, AES, BWP, DSP, HANN, HOLST, ISH, KWPN, OLDBG, OS, SF, SWB, TRAK, and WESTF.
- DSP showed significantly better performance than all studbooks except AA, AES, AWHA, BWP, CDE, HANN, HOLST, ISH, KWPN, MASAF, OLDBG, OS, PZHK, SBS, SF, SHBGB, SWB, TRAK, WESTF, and ZANG.
- AES showed significantly better performance than all studbooks except AA, AECCAá, ATA, AWHA, BWP, CDE, ChS, DSP, DWP, HANN, HOLST, ISH, KWPN, MASAF, NZWB, OLDBG, OS, PZHK, RHEIN, SBS, SF, SHBGB, SWB, TRAK, WESTF, and ZANG.
- OLDBG showed significantly better performance than AHHA, AHS, AWÖ, BH, BHHS, BWBS, CSHA, CZEWB, ESH, FWB, HSH, KWPN NA, MECKL, NRPS, NZHS, SCSL, and WSI.
- BWP showed significantly better performance than AHHA, AHS, AWÖ, BWBS, CSHA, CZEWB, ESH, FWB, HSH, KWPN NA, and WSI.
- WESTF showed significantly better performance than AHHA, AHS, AWÖ, BWBS, CZEWB, ESH, FWB, HSH, and WSI.
- SWB showed significantly better performance than AWÖ, BWBS, ESH, HSH, and WSI.
- AA showed significantly better performance than ESH.

A detailed graph of the performance summary of the eventing horses according to studbook is attached in Supplementary Files.

## 4. Discussion

### 4.1. Effect of Sex

The ratio of the sexes of the best horses throughout all the disciplines studied clearly shows the numerical dominance of geldings. This fact is supported by a questionnaire survey [37]. According to this research, 50% respondents nominated a gelding as the best horse sex for jumping with the remainder being roughly divided between stallions (27.2%) and mares (22.2%), and mares were also the least preferred option for dressage.

The results of our previous study of the entire international jumping horse population also identified geldings as the largest group of horses competing [38]. We found that there were 43.98% geldings and 31.31% mares, with stallions being the least represented (24.71%) sex in the database [38]. The popularity of geldings for advanced eventing competitions (82.95%) was also confirmed [26]. And research by Whitaker et al. 2008 [39] also showed the dominance of geldings (85.71%) at an advanced level of eventing and showed that as the difficulty of the competitions increases, the number of mares represented decreases (from 31.18% at the novice level to 9.89% at an advanced level of eventing).

The higher numbers of geldings in the performance rankings of the monitored disciplines are also supported by the finding that the relative risk of an early end of jumping career was greater for mares and stallions when compared to geldings [40].

In addition to showing the prevalence of geldings, we have statistically proven the performance superiority of geldings in jumping and the dominance of geldings and stallions in eventing. Our eventing results are consistent with a study by Hanousek et al. 2020 [26] that found that in general geldings and stallions perform better in eventing than mares.

However, our finding of the dominance of geldings in jumping does not correspond with the results of other analyses, with the exception of a statistically non-conclusive study, where the effect of sex on jumping performance showed geldings were on average better than stallions and mares [41]. In an analysis of a large population of jumping horses within the FEI international database [38], stallions achieved statistically better performance compared to less successful geldings and mares. In national-level jumping competitions, stallions achieved a higher percentage of clean jumps than mares and geldings [18]. KWPN horse performance analysis results showed that stallions and geldings performed similarly and better than mares in jumping [10]. Roman-Popovici et al. 2015 [23] found no sex-related differences in jumping performance between horses of different sexes, but their analysis was limited to only 103 horses.

The inconsistent findings across these studies may be due to the different methodologies applied. The results of individual studies should therefore be interpreted in the context of their particular methodological approaches. Therefore, to verify our results, we expect a replication of the research will be necessary in future.

On the other hand, our result, that mares achieved the lowest number of performance points in all disciplines (statistically proven in jumping and eventing), corresponds with the results of other authors regardless of the methodology applied [11,42,43]. The results of the study by Duberstein and Gilkeson 2010 [19] showed that mares exhibited higher level of anxiety and lower affability than geldings. Maršálek et al. 2005 [18] demonstrated an increased likelihood of disobedience and failure to overcome obstacles in mares and, together with two other studies [44,45], mention the lack of concentration and increased sensitivity during heat, as possible causes.

Another reason for the lower performance of mares could be different physical conditions caused by sexual dimorphism.

According to Röneus et al. 1991 [46], thoroughbred stallions have a higher Type IIA/IIB ratio compared with mares, which gives them a performance advantage. However, no such differences have yet been reported between mares and geldings. Practical evidence of the importance of sexual dimorphism is included in the traditions of thoroughbred racing. In racing, fillies and mares are given a weight allowance when competing with males. The ratings files show that the median rating of mares is 10 lb lower than that of male horses [47]. It is established that this imbalance can be addressed to an extent by having a 7 lb mare allowance across the board. The aforementioned study on thoroughbreds and the official rules of racing demonstrate the impact of sexual dimorphism in racehorses. These conclusions cannot be generalized to sport horses, as their performance is based not only on muscle strength, but also on agility and quality of movement. Therefore, further research would be needed to provide a more detailed and validated assessment of the situation of sexual dimorphism in sport horses.

### 4.2. Effect of Age

The age distribution of horses in the top sport rankings varies across disciplines (Figure 1). Over 70% of dressage horses are between 11 and 15 years old, while younger horses are predominant in both other disciplines. In jumping, 75% of horses are between 9 and 13 years old, and in eventing, 70% of horses are between 8 and 13 years old.

In 2014, 82.5% of the population of the best jumping horses internationally was between 10 and 14 years old [23]. And the most represented age category in a long-term international jumping study was the group of horses aged 10–13 years old (54.07% of horses) [38].

In one of the few studies addressing the effect of age in eventing competitions, Hanousek et al. 2020 [26] report that the average age of horses in advanced competitions was 11 years.

In dressage, we did not find a statistically relevant difference in performance; only our results based on average values confirm the statistically significant result from Great Britain, that the performance of dressage horses peaks at the age of 10 [48]. The same age is also defined as the peak performance point for KWPN dressage horses [10].

The performance of horses of different ages is statistically significantly different in jumping and eventing. As expected, younger horses achieve significantly lower performance scores in both disciplines (Table 6). However, what distinguishes jumping and eventing is the course and peak of the performance curve of horses with respect to age (Figure 3 and Figure 4). The findings of several studies are consistent regarding the peak performance of jumping horses: between 10 and 13 years old [38], the peak at the age of 10 [10], and the least penalties at 12 and 13 years old [18].

Due to the limited number of scientific publications on the performance of eventing horses at an international level, discussing the findings and explaining the difference between the disciplines remains difficult.

Nevertheless, the age of the peak performance of the horses in the studied disciplines varies mainly due to different horse management, training systems and performance requirements, as well as due to incomparable environmental conditions and different risks of injury [4,40,49,50,51,52].

### 4.3. Effect of the Proportion of Thoroughbred Genes in Pedigree

As the descriptive statistics in Table 3 show, dressage horses have a lower average share of TB genes compared to the other monitored disciplines. A similar result of an average of 22.43% of TB genes was reported in an analysis of the top 100 dressage horses in the FEI international rankings [53]. This situation is partly caused by the presence of horses of breeds that excel in dressage, but their breeding has historically been carried out without the influence of thoroughbreds (LUSIT and PRE). Excluding these breeds, the average share of TB genes in the pedigree increases to 31.79%. Even after this adjustment, the difference from jumping and eventing remains substantial.

The higher proportion of TB genes in jumping horses (40.86%) identified in this study is consistent with findings by Roman-Popovici et al. 2015 [54], who found an average proportion of 34.75% in the best horses in jumping competitions. Higher values were reported in our previous study, [38], concerning the total population of jumping horses in international competitions. The standard proportion of TB genes in jumping horse pedigrees was 42.25% in 2010 and 42.07% in 2014, while it dropped to 41.61% in 2018 [38]. The average proportion of TB genes in the most successful jumping horses was 43.45% in 2009 and 42.97% in 2019 [55]. The highest average proportion of thoroughbred genes in the pedigree of horses was found in eventing (48.58%) [55]. Figure 2 illustrates the distribution of the numbers of top-ranked horses with different proportions of TB genes across the studied disciplines. The graph shows clear differences among the disciplines. The representation reflects the current situation and the importance of TB genes in the pedigree of the best sport horses in individual disciplines. The representation of horses with different proportions of TB genes in the pedigree in this figure is related to the differences in the requirements for the ideal performance traits of horses for different disciplines. The most represented groups reflect long-term specialized breeding and performance selection of sport horses. Experts [55,56] and authors of scientific articles [10,20,53,54] agree that it is necessary to maintain a certain proportion of thoroughbred genes in sport horses in order to preserve nobility, athleticism, toughness, stamina, speed and other traits. Together with the aforementioned traits, a higher proportion of TB genes is associated, according to some authors, with excessive excitability, poorer concentration, timidity and possible deterioration of dressage movement [21,57]. However, Budzynska et al. 2018 [57] only analyzed 3-year-old stallions in performance tests, and the only statistically significant deterioration was observed in horses with more than 75% TB genes. This deterioration affected only the trot, character and rideability of young stallions; the other 14 evaluated traits were not affected. More studies are needed to focus not only on the proportion of thoroughbred genes in the pedigree of sport horses, but also on the position of the thoroughbred ancestor in it and the possible impact on performance.

A statistically significant effect of the proportion of TB genes on the performance was found only in horses from the dressage ranking (Table 7). The results show that horses with a proportion of thoroughbred genes above 20% but below 50%, and also horses without thoroughbred ancestry (only Iberian breeds LUSIT and PRE), are significantly more successful. The four statistically most successful groups are also the only groups with above-average performance. Koenen et al. 1995 [16] found no significant effect of TB gene proportion on the results in jumping and dressage competitions in his analysis of KWPN horses. In contrast, the results of a large-scale study showed that horses with 12.50% to 50% thoroughbred genes in the pedigree achieved the best results in jumping and dressage [10]. Given the absence of more recent studies focusing on the performance of dressage horses from this perspective, we intend to focus on this issue in our subsequent research.

In jumping competitions and eventing, we have not proven any statistical effect of the proportion of TB genes on the performance of horses from the WBFSH rankings, but based on average values, we can confirm that the most suitable horses for jumping and eventing have a higher proportion of thoroughbred genes in their ancestry, specifically 30–50% in jumping and 40–80% in eventing.

In this regard, further studies could help to clarify the trend and the situation in eventing. So far, only studies confirming the quality of thoroughbreds in eventing have been published [29,58].

The inconclusive effect of the proportion of TB genes in jumping and eventing horses on their performance is consistent only with older jumping results [16]. However, other studies have reported conflicting results regarding jumping horses, likely due to their substantial methodological differences. Budzynska et al. 2018 [57] found that 3-year-old stallions with a percentage of TB genes between 25% and 50% achieved the best results in young horse performance tests in Poland. Meanwhile our previous research indicated that horses with a proportion of TB genes between 30% and 70% achieved statistically better results than horses without thoroughbred ancestry in the pedigree [38]. And research based on the results of national jumping competitions showed that a minimum of 50% of TB genes in the pedigree had a positive effect on the performance of horses in the Czech Republic [21].

### 4.4. Effect of Studbook Affiliation

The influence of the studbook should be analyzed, considering the specific breeding practices of sport horse breeds. Unlike traditional purebred horse breeds (Arabian, Anglo-Arabian, thoroughbred, Lipizzaner, Friesians, Lusitano, etc.), which have a closed studbook or allow only a limited influence of foreign breeds, most studbooks of sport horses are open to horses of other breeds. Therefore, targeted selective crossing occurs [59]. The offspring of horses of other breeds can be entered into most sport horse studbooks provided they meet the criteria for entry based on exterior quality and performance parameters [6,60]. From this perspective, sport horse populations do not meet the traditional definition of a breed by FAO 2012 [61]: “*A domestic animal population may be regarded as a breed, if the animals fulfil the criteria of (I) being subjected to a common utilization pattern, (II) sharing a common habitat/distribution area, (III) representing largely a closed gene pool, and (IV) being regarded as distinct by their breeders*”.

Open studbooks for sport horses, as well as advanced methods of artificial reproduction, allow the sharing of genetic material, mainly of breeding stallions, but to a lesser extent also that of mares. As an example, the most successful sires are active in several studbooks (Cornet Obolensky, Chacco-Blue, Johnson TN, Totilas, and Diarado) and likewise, the offspring of one mare can be entered in different studbooks (Godahra II, Quasibelle du Seigneur, and Cordula de Laubry). The genetic contribution of the sire and dam has been monitored and evaluated for a long time within individual disciplines and studbooks [14,62,63,64]. A follow-up step, thanks to the sharing of the most successful sires, could potentially be a worldwide meeting of representatives of studbooks associated under the WBFSH and the establishment of a common goal—the calculation of International Breeding Values. Calculating breeding values beyond a single studbook increases the likelihood of including a wider range of mares, allowing a more reliable result of the stallion’s contribution to be calculated [60,65].

Based on the above, it is clear that sport horse studbooks are organizations for grouping horses according to their desired performance criteria, without the preference for purebred breeding. In addition to specific requirements for pedigree and performance, the registration of a sport horse in a specific studbook also depends on the decision of the horse owner, as in many cases a horse meets the conditions for multiple studbooks. Therefore, studbook affiliation is considered a composite variable reflecting breeding practices, selection strategies, and management factors, rather than as a direct biological determinant. Our results should be seen in this context and it might be important to follow them up with a performance analysis that would include the sire factor instead of the studbook.

Affiliation to the studbook had the strongest effect observed in terms of statistical significance across all three equestrian disciplines. Statistically significant differences between individual studbooks are shown in the results and the distribution of their average values is shown in the figures in Supplementary Files. From all the studbooks, only six register the best horses across the disciplines of dressage, jumping, and eventing with statistical significance (BWP, DSP, HANN, KWPN, OLDBG, and WESTF)—see Table 8. Of these breeds, only HANN has a breeding goal focused on all monitored disciplines and has also adapted breeding programs for mares [66]. BWP and KWPN have breeding programs focused only on dressage and jumping horses [67,68]. DSP has a general breeding goal of a quality riding horse and does not have specialized performance tests [69]. The breeding goals of OLDBG and WESTF do not focus specifically on eventing, but performance tests allow for eventing testing [70,71].

In addition to these studbooks, whose representatives excel in all three disciplines, we evaluated several studbooks with statistically proven performance advantages of horses in jumping and eventing. These include the Anglo European Studbook—AES; Holsteiner—HOLST; Irish Sport Horse—ISH; Oldenburg-International Jumper—OS; Selle Français—SF; Swedish warmblood—SWB; and Zangersheide—ZANG. Apart from AES, SF and SWB, which also have dressage use of horses mentioned in their breeding goal, the other named studbooks have selection focused only on the performance of horses in jumping and eventing [70,72,73,74,75,76,77].

Unlike the current results of elite horses, our previous research on the total international jumping horse population only demonstrated the poorer performance of horses without pedigree and horses of non-sporting breeds, but there was no statistically significant difference between the studbooks of sport horses [38]. For verification, it will be necessary to repeat the analysis and possibly compare the entire competition populations of individual studbooks and equestrian disciplines.

It is not possible, within the scope of this article, to discuss the individual results of all the studbooks that appear in the monitored rankings. Our findings are supposed to mainly point out that the previously mentioned sharing of the most successful sires between populations does not guarantee a balanced quality of all the studbooks of sport horses. The methodological limitation of the number of horses in individual studbooks does not allow us to evaluate their representation, but statistics from the Olympic Games show the numerical superiority of some studbooks. According to the WBFSH 2024 [78], the KWPN (17.89%), SF (10.57%) and BWP (10.16%) were the most represented breeds overall at the Olympic Games 2024. In dressage, the KWPN (27.4%), OLDBG (20.55%) and HANN (17.81%) were the most numerous studbooks, in jumping BWP (18.95%), KWPN (15.79%) and SF (13.68%), and in eventing SF (16.67%), ISH (12.82%) and KWPN (11.54) were the most numerous studbooks. The statistics of the most successful studbooks are also confirmed by our previous research on jumping horses. In international jumping competitions, the most frequently represented horses were KWPN (19.26%), SF (14.09%), BWP (12.23%), HOLST (11.33%) and OLDBG (7.16%) [38]. Dressage and eventing still lack similar statistics regarding the entire international population. Therefore, only calculations within the framework of national research are evaluated.

We believe that the presented results may also be interesting in the context of the conclusions of other studies [6,60], which focused on comparing the breeding goals and performance parameters of the most important sport horse populations. These studies show that the situation in horse breeding has changed considerably over the past twenty years. And, thanks to the innovative approach of breeding organizations using advanced breeding methods based on genomic analyses, it is possible to monitor and test performance requirements in much more detail [79,80,81].

## 5. Conclusions

The results of this analysis of the performance of horses from the dressage, jumping and eventing WBFSH Rankings confirmed the diversity of the populations of the best horses in each discipline and demonstrated the different relevancy of the monitored factors in relation to the different disciplines. A common feature is the numerical dominance of geldings across the rankings of all disciplines, but other monitored criteria such as age structure, the proportion of thoroughbred genes and the representation of studbooks differentiate the individual disciplines. Our analysis shows a statistically significant effect of each of the observed factors in at least one discipline. Based on an analysis of a selected elite population of jumping and eventing horses, higher performance has been statistically proven in horses older than 10, which persists up to 15 years in show jumping and up to 17 years in eventing. The results indicate that high dressage performance is also achieved by horses with 40% to 50% thoroughbred genes in their ancestry, although they are less represented in this discipline. The most influential factor in all disciplines was studbook affiliation. The Belgian warmblood, German Sport Horse, Hanoverian, Royal Dutch Sport Horse, Oldenburg and Westphalian studbooks are represented among the significantly top-performing studbooks in all three disciplines.

Our conclusions are based on an observational study, but we evaluate horse performance based on the official FEI methodology and scoring, which is verified by more than 15 years of practice. Based on the differences and similarities shown between the most successful horses in the monitored disciplines, breeders and riders could be able to consider further options for adjusting breeding programs and procedures to increase the performance of sport horses. The conclusions of this study of a selected population cannot be generalized.

Further research based on the analysis of entire populations of horses competing in the disciplines studied will be necessary to objectively assess the influence of the observed factors. Therefore, in the future, we would like to focus on horse performance in dressage and eventing from the perspective of the entire competing population to assess the current situation and evolving breeding strategies.

## Figures and Tables

**Table 1:** The descriptive statistics of the WBFSH ranking achieved in different equestrian sports.

	N	Mean	Median	Mode	Frequency of Mode	Minimum	Maximum	SD
Dressage PP	691	1427.38	1553.00	1870.00	4.00	61.00	3076.00	701.85
Jumping PP	1488	463.82	320.00	5.00	47.00	1.00	2683.00	431.39
Eventing PP	1636	66.72	42.50	1.00	72.00	1.00	432.00	67.86

Abbreviations: SD, standard deviation.

**Table 2:** A percentage representation of horse sexes among the most successful horses in the equestrian disciplines in the WBFSH rankings.

	Dressage	Jumping	Eventing
Gelding	44.28	41.06	51.59
Mare	20.69	33.67	34.78
Stallion	35.02	25.27	13.63

**Table 3:** The descriptive statistics of the proportion of TB genes in horse pedigree achieved in the observed equestrian sports.

	N	Mean	Median	Mode	Frequency of Mode	Minimum	Maximum	SD
Dressage TB genes (%)	690	28.47	29.69	0.00	77.00	0.00	71.88	13.50
Jumping TB genes (%)	1483	40.86	40.82	Multiple	27.00	8.20	83.20	9.91
Eventing TB genes (%)	1593	48.58	46.68	43.16	25.00	9.18	99.80	16.93

Abbreviations: SD standard deviation.

**Table 4:** Results of GLM for studbook, sex, age, proportion of TB genes and sport season factors.

	Dressage		Jumping		Eventing	
	F	Sig.	F	Sig.	F	Sig.
Sex	0.5633	0.5696	4.5313	0.0104	4.6969	0.0093
Age	0.6624	0.7882	3.3065	0.0001	2.6483	0.0004
% TB genes	3.4809	0.0011	0.6589	0.7281	1.0387	0.4064
Studbook	39.4119	0.0000	88.2679	0.0000	56.3760	0.0000
Sport season	11.4187	0.0000	46.4796	0.0000	32.2404	0.0000

Abbreviations: F, statistics; Sig., significance.

**Table 5:** Average performance point values based on sex comparison.

	Dressage	Jumping	Eventing
Geldings	1402.00	523.95	77.32
Mares	1365.30	407.63	52.81
Stallions	1502.10	447.21	71.81

**Table 6:** The results of the Scheffé test for the effect of age in the observed disciplines.

	Significant Differences (*p* ˂ 0.01)	Best Performance	Worst Performance
Dressage	NS	10 yo	19 and 21 yo
Jumping	7 yo and 9 yo ˂ 10–15 yo 8 yo ˂ 10–16 yo	11 yo	7 and 19 yo
Eventing	6 yo and 7 yo ˂ 10–17 yo 8 yo ˂ 11–16 yo 9 yo ˂ 12–15 yo	15 yo	6, 7 and 21, 22 yo

Abbreviations: NS, not significant.

**Table 7:** The results of the Scheffé test for the effect of proportion of TB genes (in %) in the observed disciplines.

	Significant Differences (*p* ˂ 0.01)	Best Performance	Worst Performance
Dressage	Up to 20 and up to 60 groups ˂Up to 10 and up to 30, 40, 50 groups	Up to 50	Up to 60
Jumping	NS	Up to 50	Up to 10
Eventing	NS	Up to 60	Up to 10

Abbreviations: NS, not significant.

**Table 8:** The average performance points of individual studbooks in the observed disciplines.

Dressage	Points	Jumping	Points	Eventing	Points
KWPN	2343.06	BWP	1211.69	ISH	208.89
OLDBG	2168.81	SF	1210.69	HOLST	195.28
HANN	2138.08	KWPN	1194.39	SF	193.36
WESTF	2106.31	ZANG	1109.06	HANN	168.06
DWB	1956.75	HOLST	1088.25	KWPN	167.97
DSP	1846.11	OS	951.28	SHBGB	150.25
LUSIT	1732.39	WESTF	906.50	DSP	128.89
BWP	1513.61	HANN	865.33	AES	123.39
		DSP	825.61	OLDBG	114.42
		ISH	794.19	BWP	102.94
		SBS	747.42	WESTF	100.89
		SWB	720.14	SWB	96.25
		AES	702.03	AA	93.78
		OLDBG	695.22	ZANG	88.28
		RHEIN	546.19	TRAK	86.49
				OS	73.92
				PZHK	72.28

Abbreviations: AA, Anglo-Arabian; AES, Anglo European Studbook; BWP, Belgian warmblood; DSP, German Sport Horse; DWB, Danish Warmblood; HANN, Hanoverian; HOLST, Holsteiner; ISH, Irish Sport Horse; KWPN, Royal Dutch Sport Horse; LUSIT, Lusitano; OLDBG, Oldenburg; OS, Oldenburg-International Jumper; PZHK, Polish Sport Horse; RHEIN, Rhenish Warmblood; SBS, Belgian Sport Horse; SF, Selle Français; SHBGB, British Sport Horse; SWB, Swedish warmblood; TRAK, Trakehner; WESTF, Westphalian; ZANG, Zangersheide. Repeating studbooks between disciplines are highlighted in color.

## Data Availability

The original data presented in the study are openly available on the WBFSH website https://wbfsh.com/ and on the FEI website https://fei.org/. The proportion of thoroughbred genes was calculated according to HorseTelex Pedigree www.horsetelex.com. The data sets presented in this article are not readily available because data are part of an ongoing study that follows up on this research and is based on this data set. Requests to access the data sets should be directed to correspondence author.

## Supplementary Materials

The following supporting information can be downloaded at: https://www.mdpi.com/article/10.3390/ani16101509/s1, Figure S1: Performance summary of dressage horses according to studbook; Figure S2: Performance summary of jumping horses according to studbook; Figure S3: Performance summary of eventing horses according to studbook.

## Abbreviations

The following abbreviations are used in this manuscript:

AA	Association Nationale Anglo-Arabe
AAFE	Asociacion Argentina de Fomento Equino
AES	Anglo European Studbook
AHHA	American Holsteiner Horse Association
AHS	The American Hanovarian Society
ATA	American Trakehner Association
AWHA	Australian Warmblood Horse Association
AWÖ	Arbeitsgemeinschaft für Warmblutzucht in Österreich
BE/SIES	Breeders Elite Studbook for Irish & European Sporthorses
BH	Associação Brasileira de Criadores do Cavalo de Hipismo
BHHS	The British Hanoverian Horse Society
BWBS	Warmblood Breeders Studbook
BWP	Belgian Warmblood Studbook
CCDM	Criadores de Caballos Deportivos Mexicanos
CDE	Asociación Nacional de Criadores del Caballo de Deporte Español
CSHA	Canadian Sport Horse Association
CZEWB	Czech Warmblood Breeders Society
DSP	German Sporthorse Association
DWB	Danish Warmblood Society
ESH	Estonian Sport Horse Breeders Society
FEI	International Federation for Equestrian Sports
FWB	The Finnish Horse Breeding Association
GLM	Generalized Linear Model
HANN	Hannoveraner Verband
HOLST	Verband der Züchter des Holsteiner Pferdes
HSH	Hungarian Sport Horse Studbook
CH	Sportpferde Swiss Warmblood
ChS	Studbook Cheval Suisse
ISH	Irish Sport Horse Studbook
ISR-OLD NA	International Sporthorse Registry Oldenburg Registry North America
KWPN	Royal Warmblood Studbook of the Netherlands
KWPN NA	KWPN of North America
LUSIT	Associação Portuguesa De Criadores do Cavalo Puro Sangue Lusitano
MASAF	Italian Sport Horse Studbook/Ministero dell’agricoltura, della sovranità alimentare e delle forest
MECKL	Verband der Pferdezüchter Mecklenburg-Vorpommern
NRPS	Nederlands Rijpaarden en Pony Stamboek
NWB	Norwegian Warmblood Association
NZHS	New Zealand Hanoverian Society
NZWB	New Zealand Warmblood Association
OLDBG	Verband der Züchter des Oldenburger Pferdes
OS	Springpferdezuchtverband Oldenburg-International
PRE	National Purebred Spanish Breed
PSH	Portuguese Sport Horse Studbook
PZHK	Polish Horse Breeders Association
RHEIN	Rheinisches Reitpferd im Hannoveraner Verband
SAWHS	South African Warmblood Horse Society
SBS	Studbook sBs, Le Cheval de Sport Belge
SCSL	Studbook du Cheval de Selle Luxembourgeois
SF	Stud Book du Cheval Selle Français
SHBGB	Sport Horse Breeding of Great Britain
SLS	Studbook La Silla
SSW	Studbook for Slovenian Warmblood Horses
SWB	Swedish Warmblood Association
TB	Thoroughbred
TRAK	Verband der Züchter und Freunde des Ostpreussischen Warmblutpferdes Trakehner Abstammung
WBFSH	World Breeding Federation for Sport Horses
WESTF	Westfälisches Pferdestammbuch
WSI	Warmblood Studbook of Ireland
ZANG	Studbook Zangersheide
ZfDP	Zuchtverband für Deutsche Pferde
