# Supplementary figures and images for: A Comparison of the Performance of Horses from the International WBFSH Rankings in Dressage, Jumping and Eventing Based on Their Sex, Age, Proportion of Thoroughbred Genes and Affiliation to the Studbook
Source: Animals (Basel). 2026 May 14;16(10):1509. doi: 10.3390/ani16101509 (PMC13203135; doi:10.3390/ani16101509)

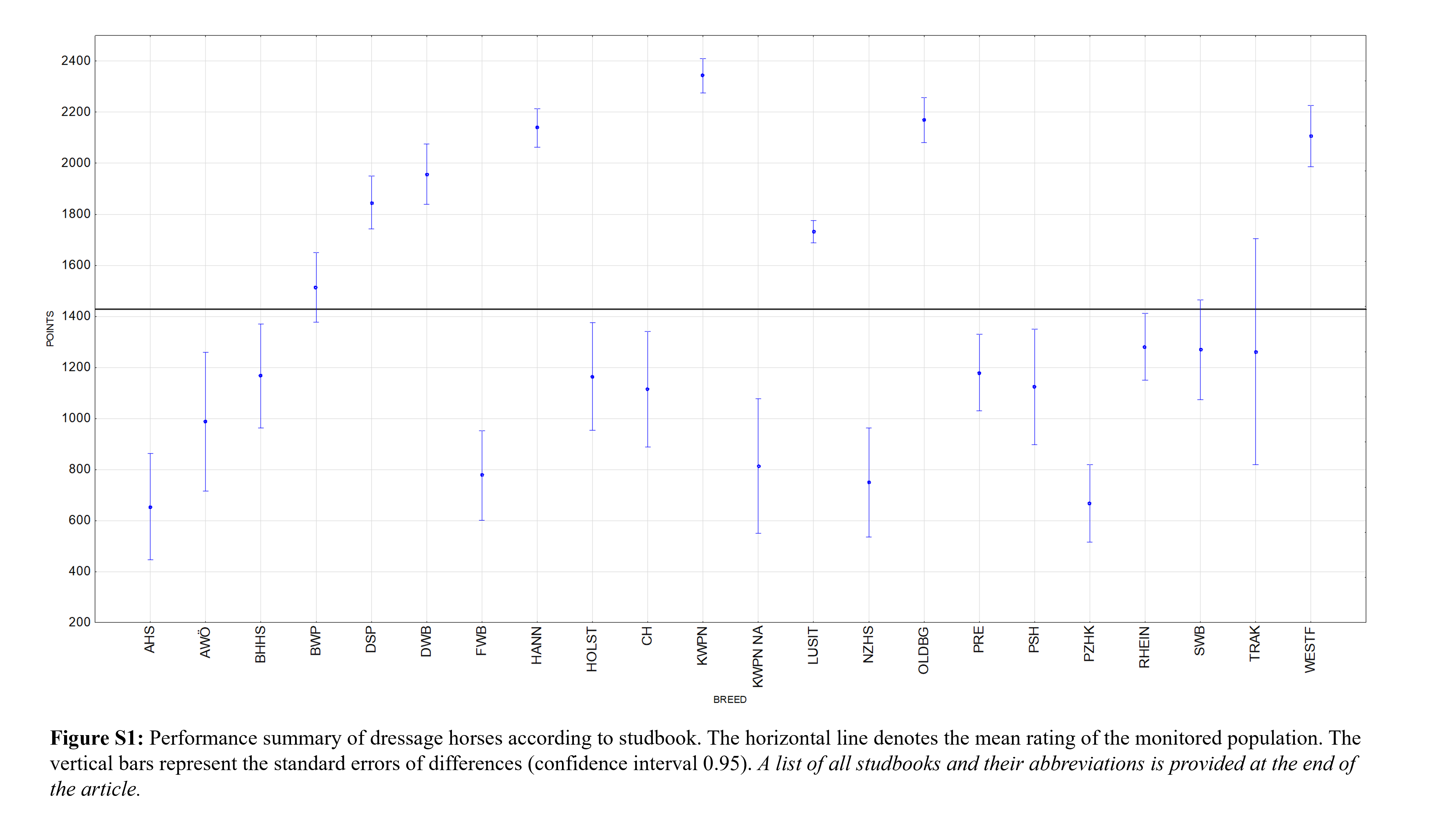

Supplement: Supplementary file 1 [file animals-16-01509-s001.zip › Figure S1 Performance summary of dressage horses according to studbook.png]

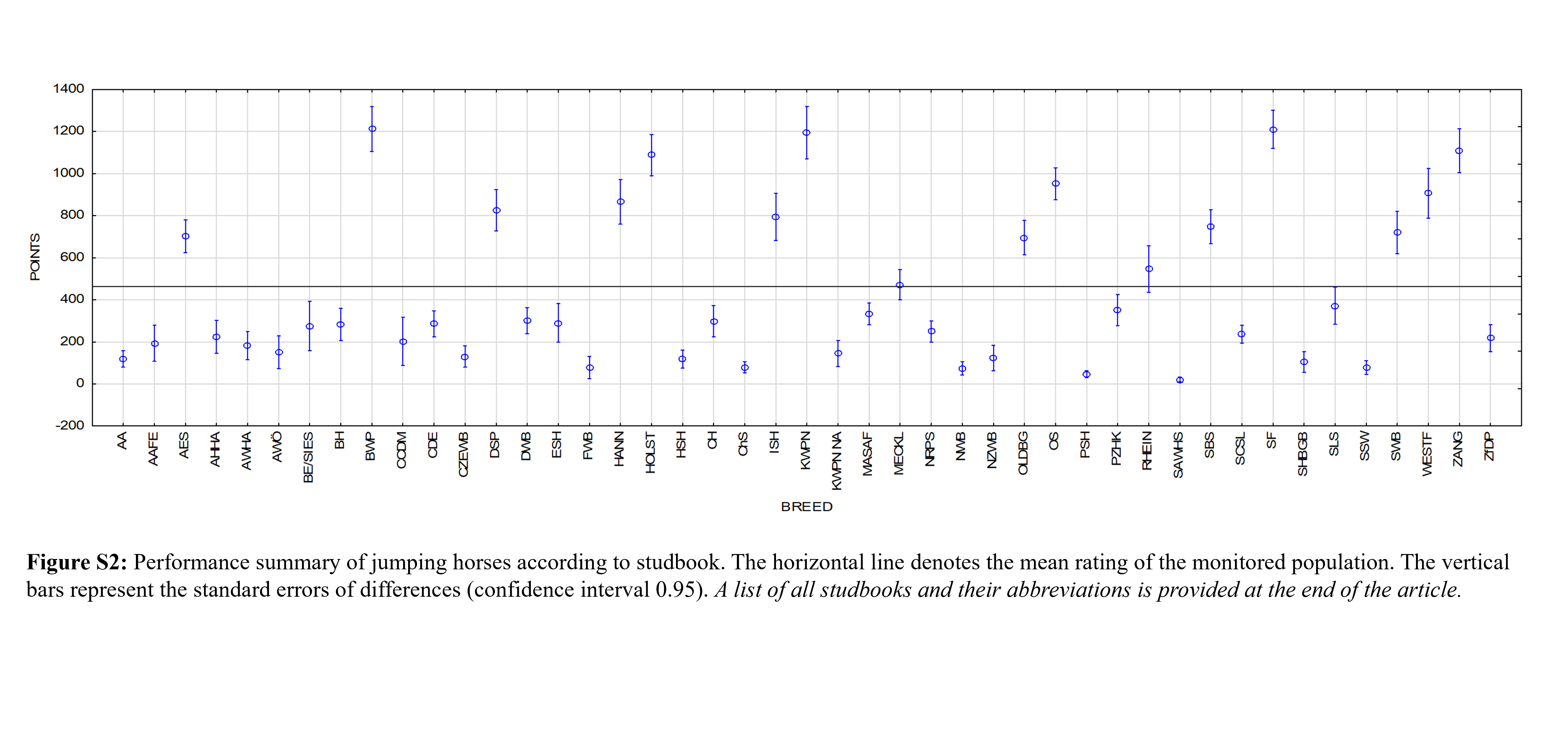

Supplement: Supplementary file 1 [file animals-16-01509-s001.zip › Figure S2 Performance summary of jumping horses according to studbook.png]

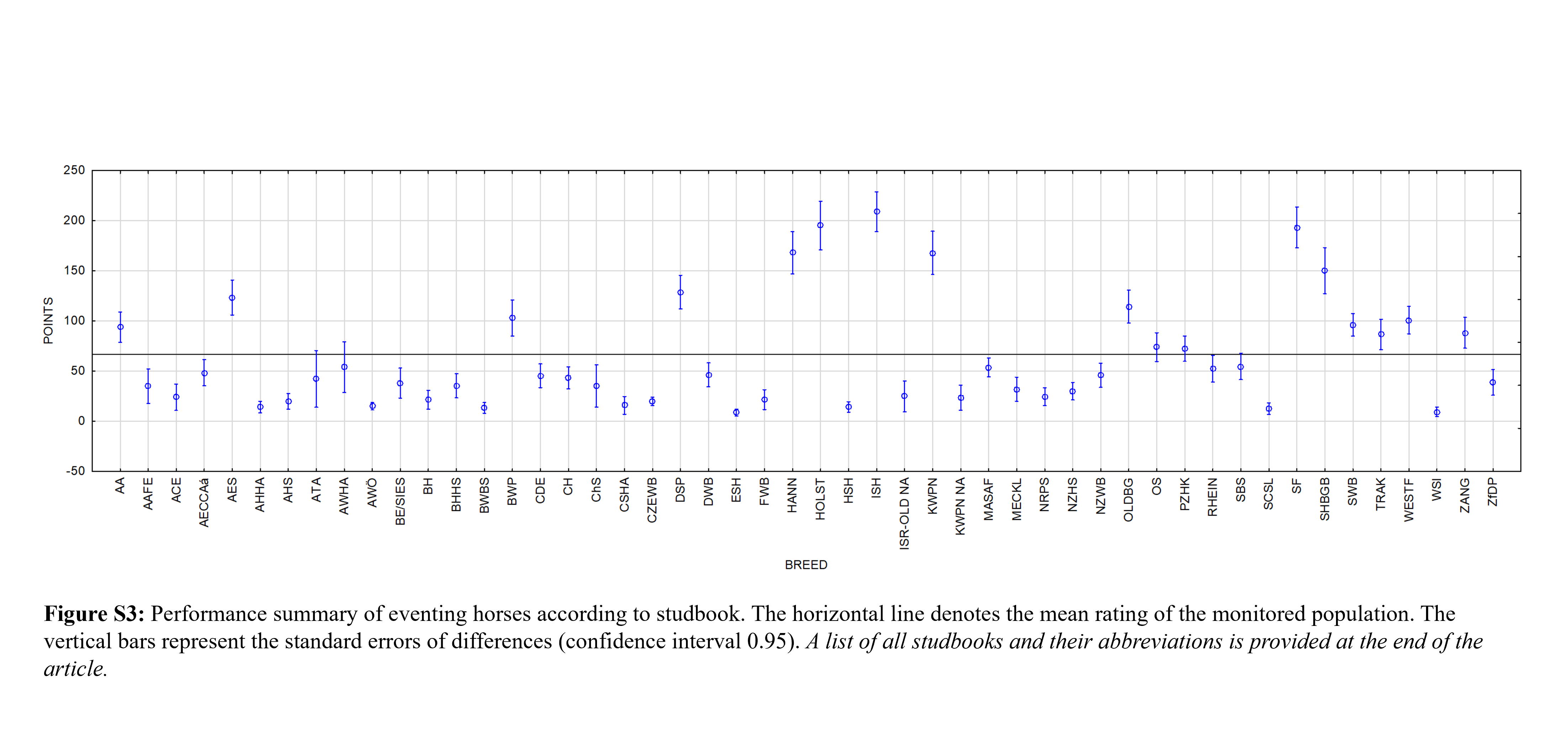

Supplement: Supplementary file 1 [file animals-16-01509-s001.zip › Figure S3 Performance summary of eventing horses according to studbook.png]
